# Disparities in breast cancer tumor characteristics, treatment, time to treatment, and survival probability among African American and white women

**DOI:** 10.1038/s41523-018-0059-5

**Published:** 2018-03-20

**Authors:** Kevin Chu Foy, James L. Fisher, Maryam B. Lustberg, Darrell M. Gray, Cecilia R. DeGraffinreid, Electra D. Paskett

**Affiliations:** 1 College of Nursing, The Ohio State University, Columbus, USA; 2 Comprehensive Cancer Center, The Ohio State University, Columbus, USA; 3 Arthur G. James Cancer Hospital and Richard J. Solove Research Institute, Columbus, USA; 4 Division of Epidemiology, College of Public Health, The Ohio State University, Columbus, USA; 5 Division of Medical Oncology, Department of Internal Medicine, College of Medicine, The Ohio State University, Columbus, USA; 6 Stefanie Spielman Comprehensive Breast Center, The Ohio State University Wexner Medical Center, Columbus, USA; 7 Division of Gastroenterology, Hepatology & Nutrition, Department of Internal Medicine, College of Medicine, The Ohio State University, Columbus, USA; 8 Division of Cancer Prevention and Control, Department of Internal Medicine, College of Medicine, The Ohio State University, Columbus, USA

## Abstract

African American (AA) women have a 42% higher breast cancer death rate compared to white women despite recent advancements in management of the disease. We examined racial differences in clinical and tumor characteristics, treatment and survival in patients diagnosed with breast cancer between 2005 and 2014 at a single institution, the James Cancer Hospital, and who were included in the Arthur G. James Cancer Hospital and Richard J. Solove Research Institute Cancer Registry in Columbus OH. Statistical analyses included likelihood ratio chi-square tests for differences in proportions, as well as univariate and multivariate Cox proportional hazards regressions to examine associations between race and overall and progression-free survival probabilities. AA women made up 10.2% (469 of 4593) the sample. Average time to onset of treatment after diagnosis was almost two times longer in AA women compared to white women (62.0 days vs 35.5 days, *p* < 0.0001). AA women were more likely to report past or current tobacco use, experience delays in treatment, have triple negative and late stage breast cancer, and were less likely to receive surgery, especially mastectomy and reconstruction following mastectomy. After adjustment for confounding factors (age, grade, and surgery), overall survival probability was significantly associated with race (HR = 1.33; 95% CI 1.03–1.72). These findings highlight the need for efforts focused on screening and receipt of prompt treatment among AA women diagnosed with breast cancer.

## Racial disparity: African Americans face delayed treatment

African Americans with breast cancer wait longer to get treated and then live shorter than white women, a US cancer center’s records show. Electra Paskett and her colleagues from Ohio State University in Columbus examined racial differences in tumor characteristics and patient outcomes among the 4,593 women treated for breast cancer at their institution’s affiliated hospitals between 2005 and 2014. They found that the time between diagnosis and treatment onset was longer for African Americans — 62 days compared to 35.5 days for white women. African Americans were also more likely to have harder-to-treat forms of disease and they were less likely to undergo surgery. Even accounting for many of these factors, African American women still had worse outcomes, as measured by survival probability. The findings highlight the need address racial disparities in breast cancer treatment.

## Introduction

In 2017, breast cancer remains the most commonly diagnosed cancer (29%) in women in the United States.^1^ Since 2004, incidence rates have been relatively stable after a 7% decrease was witnessed between 2002 and 2003.^2^ It is expected that in 2017 about 41,070 women will die from breast cancer while almost 255,180 new cases will be diagnosed.^1^ African American (AA) women have a 42% higher breast cancer death rate compared to white women.^3^ Despite recent advancements in diagnosis and treatment of breast cancer, which have significantly lowered the mortality rates in white women, AA women continue to have the highest mortality rates of the disease.^4,5^ The disparity in incidence and mortality between AA women and white women is partly due to a significant improvement in survival among white women that is lacking in AA women.^6,7^

A study by DeSantis et al.^8^ showed that, between 1975 and 2004, there was a decrease in the breast cancer mortality rate in white women in all 50 states while, among AA women, the rate decreased in only 11 states and increased substantially in two states. Previous reports have shown that, in addition to mortality and survival disparities, AA women are more commonly diagnosed at a younger age with around 33% being younger than 50 years compared to only 22% of white women;^9^ further, AA women are twice as likely to be diagnosed with breast cancer below the age of 35 when compared to white women.^10^ A higher percentage of AA women are diagnosed with late-stage disease, compared to white women, and fewer AA women have smaller (<2 cm) tumors.^10,11^

The purpose of this study was to examine disparities, among women diagnosed and/or treated at a single institution, the James Cancer Hospital, between AA and white women based on tumor and clinical characteristics (tumor receptor expression, stage at diagnosis, grade, lymph node involvement), treatment (e.g., surgery, radiotherapy therapy, chemotherapy, adjuvant hormone therapy and prophylactic mastectomy), time to treatment, overall survival (OS), and progression-free survival (PFS) probability.

## Results

### Demographics, tobacco and alcohol use history, and time to treatment onset

As shown in Table 1, the Arthur G. James Cancer Hospital and Richard J. Solove Research Institute Cancer Registry included 469 AA and 4124 white women diagnosed with breast cancer from 2005 to 2014. AA women and white women had identical mean ages at diagnosis. Significantly more AA women were not married and reported past or current tobacco use, while significantly more white women reported past or current alcohol use. Mean number of days between diagnosis and treatment was significantly greater for AA women; further, the proportion of AA women with more than 90 days between diagnosis and treatment onset was significantly greater than that for white women.

**Table 1:** Patient demographics and time to onset of treatment among African American and white breast cancer among patients treated at the James Cancer Hospital from 2005 to 2014

	White *N* (%)	African American *N* (%)	*p*-value
	4124 (89.8)	469 (10.2)	
Age at diagnosis			
Mean (years)	57.4	57.4	NS
≤40	398 (9.6)	48 (10.2)	
41–50	857 (20.8)	99 (21.1)	
51–64	1718 (41.7)	186 (39.7)	
≥65	1151 (27.9)	136 (29.0)	
Marital status at diagnosis			
Not married	1289 (31.3)	307 (65.5)	*p* < 0.0001
Married	2746 (66.5)	145 (30.9)	
Unknown	89 (2.2)	17 (3.6)	
Tobacco use history			
Past/Current use	1432 (34.7)	186 (39.7)	*p* < 0.0001
Never used	2310 (56.0)	216 (46.0)	
Unknown	382 (9.3)	67 (14.3)	
Alcohol use history			
Past/Current Use	1833 (44.5)	141 (30.1)	*p* < 0.0001
Never Used	1792 (43.5)	244 (52.0)	
Unknown	499 (12.1)	84 (17.9)	
Time to treatment onset			
Mean (days)	35.5	62.0	*p* < 0.0001
≤30	1713 (41.5)	174 (37.1)	
31–60	1480 (35.9)	147 (31.3)	
61–90	321 (7.8)	40 (8.5)	
>90	610 (14.8)	108 (23.1)	

### Tumor characteristics and lymph node involvement

As shown in Table 2, a significantly greater proportion of AA women were diagnosed with triple negative breast cancer, and a significantly greater proportion of AA women were diagnosed with advanced (regional and distant) stages of breast cancer. No significant differences by race were observed for grade. There were significantly greater proportions of AA women with both positive and unknown lymph node involvement compared to white women.

**Table 2:** Receptor status, stage at diagnosis, grade, and lymph node involvement among African American and white breast cancer patients treated at the James Cancer Hospital from 2005 to 2014

	White *N* (%) 4124 (89.8)^a^	African American N* (%) 469 (10.2)^a^	*p*-value
*Receptor status*			
Hormone receptor positive/HER2 negative	3038 (71.2)	286 (59.1)	*p* < 0.0001
HER2 positive	382 (9.0)	48 (9.9)	
Triple negative	845 (19.8)	150 (31.0)	
*Stage at diagnosis*			
In situ	1866 (45.3)	158 (33.7)	*p* < 0.0001
Localized	1489 (36.1)	179 (38.2)	
Regional	487 (11.8)	88 (18.8)	
Distant	282 (6.8)	44 (9.4)	
*Grade*			
I	791 (19.8)	96 (20.5)	NS
II	1607 (39.0)	188 (40.1)	
III	1456 (35.3)	153 (32.6)	
IV	3 (0.1)	2 (0.4)	
Unknown	267 (6.5)	30 (6.4)	
*Lymph node involvement*			
0	2417 (58.6)	222 (47.3)	*p* < 0.0001
≥1	1317 (31.9)	163 (34.8)	
Unknown	390 (9.5)	84 (17.9)	

^a^Note that sample sizes do not always add to the total and percents do not always add to 100.0% due to missing data and rounding

### Treatment

As shown in Table 3, hormonal therapy, among those with hormone receptor positive breast cancer, was significantly more common among AA women. Surgery was significantly less common among AA women, compared to white women. Specifically, mastectomy, reconstruction after mastectomy, and prophylactic mastectomy and reconstruction in the unaffected breast were significantly less common among AA women. Although breast-conserving surgery was less common among AA women, the difference was not statistically significant. Table 4 shows treatment according to stage at diagnosis. Significantly fewer AA women received radiotherapy for those diagnosed with regional stage breast cancer, and breast-conserving surgery for those diagnosed with in situ and localized stage disease. Among those diagnosed with hormone receptor positive breast cancer at the in situ and localized stages, significantly more AA women received hormonal therapy. Further, among those diagnosed with regional and distant stage disease, significantly more AA women received immunotherapy. Last, among those diagnosed with localized breast cancer, significantly more AA women received neoadjuvant therapy.

**Table 3:** Treatment among African American and white breast cancer patients treated at the James Cancer Hospital from 2005 to 2014

	White *N* (%) 4124 (89.8)^a^	African American *N* (%) 469 (10.2)^a^	*p*-value
*Treatment*			
Chemotherapy	2291 (53.7)	273 (56.4)	NS
Hormonal therapy (among those hormone receptor positive)	2235 (68.1) (out of 3280)	244 (78.0) (out of 313)	0.0003
Radiotherapy	2276 (55.2)	252 (53.7)	NS
Immunotherapy	359 (8.7)	42 (9.0)	NS
Neoadjuvant therapy	576 (14.0)	69 (14.7)	NS
Surgery	3871 (93.8)	424 (90.4)	0.0251
*Surgery type (among those receiving surgery)*			
Mastectomy	1674 (40.6)	169 (36.0)	*p* = 0.033
Reconstruction (among those receiving mastectomy)	936 (55.9)	86 (50.9)	*p* = 0.032
Breast-conserving surgery	1971 (47.8)	214 (45.6)	NS
Radiotherapy (among those receiving breast conserving surgery)	1971 (100.0)	213 (99.5)	NS
Prophylactic mastectomy and reconstruction in unaffected breast	461 (11.2)	37 (7.9)	*p* = 0.050

^a^Note that sample sizes do not always add to the total and percents do not always add to 100.0% due to missing data and rounding

**Table 4:** Treatment according to stage at diagnosis among African American and white breast cancer patients treated at the James Cancer Hospital from 2005 to 2014

	In situ *N* (%)		Localized *N* (%)		Regional *N* (%)		Distant *N* (%)	
Treatment^a^	White 1866 (45.3)	Black 158 (33.7)	White 1489 (36.1)	Black 179 (38.2)	White 487 (11.8)	Black 88 (18.8)	White 282 (6.8)	Black 44 (9.4)
Chemotherapy	558 (29.9)	45 (28.7)	1093 (73.4)	126 (70.4)	446 (91.6)	78 (88.6)	186 (66.1)	32 (72.7)
Hormonal therapy (among those hormone receptor positive)	**1100 (68.6)**	**101 (82.8)**	**751 (68.6)**	**87 (83.7)**	250 (69.8)	41 (73.2)	134 (59.8)	15 (48.4)
Radiotherapy	1009 (54.1)	80 (51.0)	752 (50.5)	96 (53.6)	**410 (84.2)**	**62 (70.5)**	104 (36.8)	15 (34.1)
Immunotherapy	87 (4.7)	11 (7.0)	150 (10.1)	18 (10.1)	**73 (15.0)**	**20 (22.7)**	**47 (16.8)**	**13 (29.5)**
Neoadjuvant therapy	82 (4.4)	6 (3.8)	**266 (17.9)**	**57 (31.8)**	135 (27.8)	26 (29.5)	27 (9.6)	13 (29.5)
Surgery	1828 (98.0)	148 (93.6)	1463 (98.3)	168 (93.8)	475 (97.5)	81 (92.0)	251 (89.0)	38 (86.4)
Breast-conserving surgery	**762 (40.8)**	**56 (35.4)**	**670 (45.0)**	**74 (41.3)**	215 (44.1)	41 (46.6)	108 (38.3)	21 (47.7)
Radiotherapy (among those receiving breast-conserving surgery)	609 (79.9)	44 (78.6)	524 (78.2)	60 (81.1)	173 (80.5)	35 (85.4)	85 (78.7)	17 (81.0)

^a^Bolded numbers and percentages represent statistically significant Black-White differences. Note that sample sizes do not always add to the total and percents do not always add to 100.0% due to missing data and rounding

### Overall survival and progression-free survival

The mean follow-up period for AA women (1734 days) was significantly shorter than the mean follow-up period for white women (1926 days). Table 5 shows results from univariate and multivariate regressions. Overall survival probability and PFS was lower among AA women, compared to white women (HR = 1.34; 95% CI 1.05–1.70; HR = 1.33; 95% CI 1.07–1.6, respectively). Additional factors associated with higher OS probability in univariate regressions were: younger age at diagnosis, married marital status, past or current alcohol use, late stage at diagnosis, undifferentiated or poorly differentiated grade, triple negative status, receipt of any type of surgery, receipt of radiotherapy, receipt of chemotherapy, receipt of hormonal therapy (among women with hormone positive breast cancer), and longer (31–60 days) time to treatment. Additional factors associated with higher PFS probability in univariate regressions were: past or current alcohol use, undifferentiated or poorly differentiated grade, triple negative status, receipt of any type of surgery, receipt of radiotherapy, receipt of chemotherapy, receipt of hormonal therapy (among women with hormone positive breast cancer), and longer (31–60 days) time to treatment. Factors remaining in final models for both OS and PFS (each model developed independently), other than race, were: age at diagnosis, undifferentiated or poorly differentiated grade, and receipt of any type of surgery. Adjustment for confounding by additional factors did not substantively alter, or remove the statistically significant impact, of AA race on OS probability. Adjustment for confounding by age, grade and surgery attenuated the impact, and removed the statistical significance of AA race on PFS probability. Time to treatment was not statistically significant in final models of OS and PFS probability and time to treatment did not substantively alter the impact of AA race on OS and PFS probability. It should be noted that it was possible for a factor to be strongly associated with survival probability and not be in the final model, if the factor did not alter the association between race and survival probability (e.g., stage at diagnosis).

**Table 5:** Hazard ratios and 95% confidence intervals reflecting univariate and multivariate Cox proportional hazards regression models of associations between race and overall and progression-free survival probability among breast cancer patients treated at the James Cancer Hospital from 2005 to 2014

	Overall survival	Progression-free survival
	Hazard ratio (95% CI)	Hazard ratio (95% CI)
*Univariate regressions*		
African American race (ref: white)	1.34 (1.05–1.70)	1.33 (1.07–1.65)
Age at diagnosis (per year)	1.00 (1.00–1.00)	1.00 (1.00–1.00)
Married marital status (ref: unmarried)	0.90 (0.76–1.06)	0.88 (0.76–1.03)
Past or current tobacco use (ref: no use)	0.99 (0.84–1.16)	1.08 (0.94–1.26)
Past or current alcohol use (ref: no use)	0.81 (0.69–0.95)	0.85 (0.74–0.98)
Regional and distant stages at diagnosis (ref: in situ and localized)	1.10 (1.03–1.22)	1.14 (0.95–1.36)
Poorly differentiated or undifferentiated grade (ref: well and moderately differentiated)	1.74 (1.47–2.05)	1.72 (1.48–2.01)
Triple negative (ER-/PR-/HER2-; ref: other hormone receptor and HER2 status combinations)	1.49 (1.24–1.79)	1.61 (1.38–1.89)
At least 1 lymph node involved (ref: no nodes involved)	1.13 (0.95–1.35)	1.13 (0.96–1.32)
Surgery (ref: no surgery)	0.36 (0.29–0.44)	0.29 (0.24–0.34)
Radiotherapy (ref: no radiotherapy)	0.83 (0.71–0.98)	0.75 (0.65–0.86)
Chemotherapy (ref: no chemotherapy)	1.31 (1.11–1.54)	1.19 (1.02–1.38)
Hormonal therapy (ref: no hormonal therapy)	0.69 (0.59–0.81)	0.61 (0.52–0.70)
*Time to treatment*		
≤30 days (ref.)		
31–60 days	4.32 (1.72–10.83)	3.51 (1.43–8.61)
61–90 days	1.24 (0.30–5.11)	1.32 (0.42–4.16)
>90 days	1.44 (0.52–4.00)	1.03 (0.38–2.79)
*Multivariate model*		
African American race (ref: white)	1.33 (1.03–1.72)	1.20 (0.95–1.52)
Age at diagnosis (per year)	1.00 (1.00–1.00)	1.00 (1.00–1.00)
Poorly differentiated or undifferentiated grade (ref: well and moderately differentiated)	1.78 (1.50–2.11)	1.72 (1.47–2.00)
Surgery (ref: no surgery)	0.34 (0.27–0.41)	0.28 (0.23–0.34)

## Discussion

The aim of this study was to assess the disparities between AA women and white women diagnosed with breast cancer at a single institution, The James Cancer Hospital, between 2005 and 2014. AA women were more likely to report past or current tobacco use, experience delays in treatment, have triple negative and late stage breast cancer, and were less likely to receive surgery, especially mastectomy and reconstruction following mastectomy. After adjustment for confounding factors, AA women had significantly lower OS probability.

Factors that may contribute to disparities in risk of death from breast cancer and associated survival probability include social and behavioral factors, screening behavior disparities, and differences in tumor biology and treatment. Tobacco smoking increases risk of death from breast cancer.^12^ In the present study, AA women were more likely to report current or past tobacco use. AA women were also more likely to be unmarried and partner availability may affect breast cancer survival outcomes with married or partnered couples showing improved prognosis, possibly attributable to increased social support.^13^ A higher prevalence of divorce and separation, as well as the associated psychosocial stress, experienced by AA women may also contribute to the observed lower survival probability.

Screening behavior and access to screening may contribute to disparities in risk of death from breast cancer and survival probability. Indeed, we observed that AA women were more likely to be diagnosed with advanced stages of breast cancer and to have lymph node involvement. No screening information was available in the present study. However, other studies have reported that AA women and other minority groups are less likely to visit centers with digital mammography and with designated imaging experts to read the films.^14,15^

Differences in tumor biology may also contribute to the race disparity in risk of death from breast cancer and survival probability. Triple negative breast cancer is more common among AA women, and AA women, irrespective of age and stage at diagnosis, have been found to present with more grade III tumors, while white women were more likely to present with grade I and II tumors;^16,17^ however, these patterns in grade were not observed in the present study.

The racial disparity in breast cancer survival may also be partially attributable to differences in patterns of care. Even after adjustment for age, stage at diagnosis, receptor expression status and socioeconomic status, AA women have been found to remain twice as likely as white women to die from breast cancer.^18^ We found that significantly fewer AA women received surgery overall, and were less likely to receive breast-conserving surgery among those with in situ and localized breast cancers. AA women were significantly more likely to receive hormonal therapy which may prevent cancer recurrence in women diagnosed with early stage hormone receptor positive breast cancer.^19^ There were also disparities in radiotherapy (among those with regional stage disease) and in mastectomy, reconstruction after mastectomy, and prophylactic mastectomy. For all stages combined, although breast-conserving surgery was less common among AA women, the difference was not statistically significant.

Overall, white women had higher OS and PFS, both after adjustment for confounding factors (only statistically significant for OS). Although significant differences in time to treatment were observed between AA and white women, these differences were not significantly associated with OS or PFS probability after adjustment for other factors. Silber et al.^20^ showed that AA women experience significant delays in onset of treatment after breast cancer diagnosis when compared to white women and are twice as likely to wait for more than three months before initiating treatment. Results from the present study confirm this finding; moreover, more AA women in this study waited for more than 90 days before initiating treatment, similar to findings previously reported.

The study was not without limitations. First, only women diagnosed with breast cancer at The James Cancer Hospital were included. As a result, the findings reported here may not be generalizable to populations outside of (largely Central) Ohio, the Midwestern United States, and/or those treated at a Comprehensive Cancer Center. However, the limitation observed in the generalizability of this single institution study may be somewhat balanced by the fact that many results reported in other investigations have been confirmed in one group of patients at one institution, in which there is likely less variation in potentially confounding factors such as institution-based screening, diagnosis and treatment patterns. Second, we relied on information available in the Arthur G. James Cancer Hospital and Richard J. Solove Research Institute Cancer Registry and, as such, we were unable to assess factors like socio-economic status (including insurance status), residential distance from the medical center, access to transportation, obesity, and comorbid conditions; therefore, it was not possible to identify and examine the potential confounding of comorbid conditions that may have contributed to the lower survival probability observed for AA women. Indeed, it may be important to consider confounding by comorbid conditions like diabetes and cardiovascular diseases, as previously illustrated by Tammemagi et al.^21^ Third, the absence of information about loss to follow-up may have resulted in inaccurate estimates of survival probability; however, this absence is (unfortunately) standard in survival analyses of cancer registry data, including SEER data. Lastly, the mean follow-up period for AA women (1734 days) was significantly shorter than the mean follow-up period for white women (1926 days), and this difference may have biased results.

This study showed that factors contributing to breast cancer disparities are diverse and may occur as a result of interplay between causes occurring at multiple levels, from behavioral risk factors to treatment and survivorship. In order to reduce breast cancer disparities, it is paramount that we fully understand the complex interplay of key factors. This is in line with one of the main objectives of the nationwide strategy to improve health outcomes, as outlined in the Patient Protection and Affordable Care Act of 2010.^22^ As proposed by Daly et al.^11^ it is important to design interventions that can help reduce the racial disparity in outcomes between AA and white women and these interventions should follow a combination approach involving increased insurance coverage, patient education, patient navigation, and substantive changes in the health care delivery and referral system.

## Methods

### Study population and data sources

We conducted a study of data from the retrospective chart review of breast cancer patients from the Arthur G. James Cancer Hospital and Richard J. Solove Research Institute Cancer Registry.^23^ Data were obtained from 469 AA women and 4124 white women diagnosed with primary breast cancer between 2005 and 2014. All data included in this study were extracted from medical records and patients were not contacted for the purpose of this study. Due to the retrospective nature of this study of cancer registry data, it was not necessary to obtain informed consent from patients; therefore, a waiver of informed consent was obtained from the OSU Institutional Review Board.

Information about the following factors was examined: demographics, including race, age at diagnosis, and marital status; tobacco and alcohol use history; clinical factors, including tumor receptor expression, stage at diagnosis, grade, lymph node involvement, time to onset of treatment, treatment; vital status; and dates of diagnosis, treatment, recurrence, and death.

Stage at diagnosis was characterized using a summary measure with categories of in situ, localized, regional and distant stages. The grade/differentiation was classified using the system developed by the North American Association of Central Cancer Registries (NAACCR) and the Surveillance, Epidemiology and End Results (SEER) Program, which classifies tumors as well differentiated, moderately differentiated, poorly differentiated, and undifferentiated.

For OS, survival time was characterized as the number of days between date of diagnosis and either date of death or date of the end of study (May 1, 2016). For PFS, survival time was characterized as the number of days between date of diagnosis and either date of recurrence, death or date of the end of study (May 1, 2016). Cause of death was not collected by the Arthur G. James Cancer Hospital and Richard J. Solove Research Institute Cancer Registry; as a result, we were not able to examine cause-specific death rates or cause-specific survival. Vital status was determined by the Arthur G. James Cancer Hospital and Richard J. Solove Research Institute Cancer Registry using several sources of information. These included: death follow-back from the Ohio Cancer Incidence Surveillance System (OCISS), the central cancer registry for the State of Ohio, Integrated Health Information Systems (IHIS), The Social Security Death Index (SSDI), obituaries and social media. These sources of vital status information are similar to those used by the SEER Program.

### Statistical analyses

Percentages were used to describe demographic, behavioral, clinical and outcome information according to race. Likelihood ratio chi square tests were used to test for statistically significant differences between race and selected characteristics, including survival outcomes. The log-rank test was used to compare the survival probabilities. Cox proportional hazard regressions were used to examine OS and PFS while adjusting for additional relevant clinical covariates, as allowed, based on the number of events per group. Potential confounders of associations between race and both PFS and OS were those that were at least marginally significant (*p* < 0.10) in univariate regressions of associations with OS and PFS. A potential confounder remained in the final model if the factor was statistically significant and removal of the potential confounder altered the regression coefficient of the primary factor of interest (race) considerably (at least 10%). For each covariate remaining in a model, the assumption of proportional hazard functions was tested by visually examining the plot of the hazard function for each level of the factor of interest, and by testing the statistical significance of a term in the model reflecting the multiplicative interaction between the time variable and the factor of interest. (For all models subsequently presented, no substantive violations of the proportional hazards assumption were identified.) Alpha was set at 0.05 for all statistical tests. SAS software version 9.4, was used to conduct statistical analyses.^24^

### Data availability

The data that support the findings of this study are available from the Arthur G. James Cancer Hospital and Richard J. Solove Research Institute Cancer Registry, but restrictions apply to the availability of these data, which were used under license for the current study, and so are not publicly available. Data are however available from the authors upon reasonable request and with permission of the Arthur G. James Cancer Hospital and Richard J. Solove Research Institute Cancer Registry.

## References

[1] American Cancer Society. Cancer Facts & Figures 2017. (2017).

[2] Cronin KA, Ries LA, Edwards BK (2014). The surveillance, epidemiology, and end results (SEER) Program of the National Cancer Institute. Cancer.

[3] SEER Cancer Statistics Review, 1975–2014, https://seer.cancer.gov/csr/1975_2014/, based on November 2016 SEER data submission, posted to the SEER web site, April 2017.

[4] Centers for Disease, C. & Prevention. Vital signs: racial disparities in breast cancer severity—United States, 2005–2009. *MMWR Morb Mortal Wkly Rep* **61**, 922–926 (2012). 23151952

[5] Akinyemiju TF (2013). Trends in breast cancer stage and mortality in Michigan (1992–2009) by race, socioeconomic status, and area healthcare resources. PLoS One.

[6] Hayat MJ, Howlader N, Reichman ME, Edwards BK (2007). Cancer statistics, trends, and multiple primary cancer analyses from the surveillance, epidemiology, and end results (SEER) Program. Oncologist.

[7] Hunt BR, Whitman S, Hurlbert MS (2014). Increasing Black:White disparities in breast cancer mortality in the 50 largest cities in the United States. Cancer Epidemiol.

[8] DeSantis C, Jemal A, Ward E, Thun MJ (2008). Temporal trends in breast cancer mortality by state and race. Cancer Causes Control.

[9] Clarke CA (2003). Existing data on breast cancer in African-American women: what we know and what we need to know. Cancer.

[10] Kurian AW, Fish K, Shema SJ, Clarke CA (2010). Lifetime risks of specific breast cancer subtypes among women in four racial/ethnic groups. Breast Cancer Res.

[11] Daly B, Olopade OI (2015). A perfect storm: How tumor biology, genomics, and health care delivery patterns collide to create a racial survival disparity in breast cancer and proposed interventions for change. Cancer J. Clin..

[12] Wang K, Li F, Zhang X, Li Z, Li H (2016). Smoking increases risks of all-cause and breast cancer specific mortality in breast cancer individuals: a dose-response meta-analysis of prospective cohort studies involving 39725 breast cancer cases. Oncotarget.

[13] Kvikstad A, Vatten LJ, Tretli S (1995). Widowhood and divorce in relation to overall survival among middle-aged Norwegian women with cancer. Br. J. Cancer.

[14] Ansell D (2009). A community effort to reduce the black/white breast cancer mortality disparity in Chicago. Cancer Causes Control.

[15] Gehlert S (2008). Targeting health disparities: a model linking upstream determinants to downstream interventions. Health Aff..

[16] Adams SA (2012). Racial disparities in breast cancer mortality in a multiethnic cohort in the Southeast. Cancer.

[17] Henson DE, Chu KC, Levine PH (2003). Histologic grade, stage, and survival in breast carcinoma: comparison of African American and Caucasian women. Cancer.

[18] Fisher B (1989). A randomized clinical trial evaluating tamoxifen in the treatment of patients with node-negative breast cancer who have estrogen-receptor-positive tumors. N. Engl. J. Med.

[19] Gwyn K (2004). Racial differences in diagnosis, treatment, and clinical delays in a population-based study of patients with newly diagnosed breast carcinoma. Cancer.

[20] Silber JH (2013). Characteristics associated with differences in survival among black and white women with breast cancer. JAMA.

[21] Tammemagi CM, Nerenz D, Neslund-Dudas C, Feldkamp C, Nathanson D (2005). Comorbidity and survival disparities among black and white patients with breast cancer. JAMA.

[22] Kline JA, Walthall JD (2010). Patient Protection and Affordable Care Act of 2010: summary, analysis, and opportunities for advocacy for the academic emergency physician. Acad. Emerg. Med.

[23] Arthur G. James Cancer Hospital and Richard J. Solove Research Institute Cancer Registry. (2016).

[24] SAS Institute Inc., Cary, NC, USA.

